# Inequity in mortality rates and potential years of life lost caused by COVID-19 in the Greater Santiago, Chile

**DOI:** 10.1038/s41598-023-43531-x

**Published:** 2023-09-28

**Authors:** Andrés Ayala, Claudio Vargas, Felipe Elorrieta, Pablo Villalobos Dintrans, Matilde Maddaleno

**Affiliations:** 1 Departamento de Matemática y Ciencia de la Computación, Facultad de Ciencias, Universidad de Santiago de Chile, Santiago, Chile; 2 Programa Centro Salud Pública, Facultad de Ciencias Médicas, Universidad de Santiago de Chile, Santiago, Chile

**Keywords:** Public health, Epidemiology

## Abstract

Several studies have shown that, in Chile, income inequality is relevant in explaining health inequities. The COVID-19 pandemic has also had a negative impact, with higher mortality rates in those municipalities of Greater Santiago with lower socioeconomic status. We study inequity in mortality based on Potential Years of Life Lost (PYLL) in 34 urban municipalities of the Metropolitan Region (Greater Santiago) and analyze its evolution between 2018 and 2021 and by COVID-19 waves. To compare the results obtained for PYLL, we also computed the mortality rates adjusted by direct standardization. In addition, we used the concentration index (CI) to measure the health inequalities between municipalities. In the first year of the pandemic, the absolute PYLL and the standardized mortality rate for all causes of death showed an increase of 13.6% and 18.9%, respectively. Moreover, 409,086 years of life were prematurely lost in 2020, one-fifth of them due to COVID-19. The concentration indices confirm inequality in both mortality rates and PYLL, where it is more pronounced when calculating the latter measure. Results show that the deaths due to the COVID-19 pandemic affected the most economically disadvantaged municipalities, and particularly young people in those places.

## Introduction

Since its arrival, Chile has been dealing with COVID-19. After two years of stringent restrictions, the country has moved into a situation of “new normal”, in which many restrictions have been removed^1,2^. Although today the situation looks better than two years ago, COVID-19 is an ongoing crisis. By November 27, 2022, the country registered 4.9 million accumulated cases and 62,377 COVID-related deaths^3^.

Many public policies have been implemented during these years, mostly focused on reducing the number of cases and deaths, including mobility restrictions and a large-scale vaccine rollout^1,4^. These actions have improved health outcomes such as deaths and hospital bed occupancy rates^3^. Even though these are good news—reducing the overall impact of the pandemic—, fewer analyses are available on the distributional effects of COVID-19 in Chile.

Despite the existence studies on the distributional effects of the COVID-19 in other Latin American countries^5–8^, Chile emerges as a relevant case study because income inequality has been traditionally pointed as relevant to explain health inequities^9^; particularly for Greater Santiago, the COVID-19 impact has also shown a negative pattern, with worse outcomes registered in places with lower socioeconomic level^10–13^.

In this context, thinking about inequality and inequity in Chile is crucial for several reasons. First, highlighting the unequal effect of the pandemic but also the potential impacts of the policies implemented to address COVID-19 is relevant for designing new strategies, particularly in this period of “new normal” in the country. Second, a broader discussion has been carried out in the past, mostly motivated by the existence of unjust and avoidable inequalities, including those in the health system, that ended up in the social outbreak of October 2019, a new coalition in power, a process to change the constitution and, today, a debate for reforming the health system^14–16^.

The aim of this article is to measure the impact of COVID-19 on mortality inequity—over a period of 4 years—in the Greater Santiago area, comprising 34 urban municipalities of the Metropolitan Region, Chile. The Metropolitan Region is an interesting case of study, since it concentrates more than 40% of the country’s population—35.9% of the country’s population living in the Greater Santiago area—, with an important socioeconomic and demographic heterogeneity at the municipality level^17^.

To measure mortality inequity, adjusted mortality rates and potential years of life lost (PYLL) were used. The PYLL differs from adjusted mortality rates in that they consider the age of the people who died in their calculation, giving higher values if more young people have died in a given municipality. Although previous studies have examined mortality inequity in the Metropolitan Region^10,11^, none of them have focused on analyzing PYLL. PYLL has been used to study mortality inequity by COVID-19 in other countries^18–20^ and for other diseases^21–23^.

Understanding the distributional effects of the disease can help advocate for the implementation of policies targeted to those in more need, and emphasize the urgency of addressing the structural inequities in the country and in the Chilean health system.

## Methods

The study utilizes data from the 34 urban municipalities of the Metropolitan Region, known as Greater Santiago. For each municipality, two mortality measures were calculated: adjusted mortality rates and potential years of life lost (PYLL). Mortality rates are adjusted by direct standardization, i.e., they are calculated considering the same age structure per administrative unit. Thus, possible confounding effects of the composition of each population are eliminated, allowing comparison between municipalities. For the PYLL, the OECD 2022 methodology was adopted, which also standardizes by age and considers a threshold of 75 years for a premature death^24^.

The expressions for the standardized mortality rate (SMR) and potential years of life lost (PYLL) are as follows:

1 $$SM{R}_{it}={\sum }_{a=0}^{K}({d}_{at}/{P}_{at})*{Pr}_{a}*\mathrm{1,000}$$

2 $${PYLL}_{it}={\sum }_{a=0}^{L-1}(L-a)({d}_{at}/{P}_{at})*{Pr}_{a}*\mathrm{1,000}$$

$SM{R}_{it}$ is the standardized mortality rate per 1000 people using the direct method in the municipality “i” in period “t”. $K$ is the maximum age reached at the time of death in the municipality “i” in period “t”. ${d}_{at}$ is the number of deaths at age “a” in the municipality “i” in period “t”. ${P}_{at}$ is the number of persons aged “a” in the municipality “i” in period “t”. ${Pr}_{a}$ is the proportion of persons aged “a” in the 2015 OECD population (reference population^25^). ${PYLL}_{it}$ is the potential years of life lost in the municipality “i” in period “t”. L is the upper age limit established for the calculation of the measure (75 years according to the OECD).

The weighting of deaths at early ages can be observed in the term $(L-a)$ in Eq. (2). Thus, a person who dies at 15 years of age has more weight in the PYLL index than one who dies at 70 years of age, because the first contributes to 60 years of life lost prematurely while the second has only 5 years of life lost prematurely.

Additionally, the absolute potential years of life lost is reported, which is calculated at the regional level as:

3 $${ABS\_PYLL}_{jt}={\sum }_{a=0}^{L-1}(L-a){d}_{at}$$

${ABS\_PYLL}_{jt}$ is the absolute potential years of life lost for the cause of death "j" in period "t". L is the upper age limit established for the calculation of the measure (75 years). ${d}_{at}$ is the number of deaths at age “a” in period “t”.

To study the evolution of the indicators, two approaches were followed. First, yearly data were used, considering four different periods: two before the onset of the pandemic (2018 and 2019) and two to capture mortality patterns during the pandemic (2020 and 2021). Second, indicators were calculated for each COVID-19 wave. For this purpose, we used a previously defined way to identify COVID-19 waves, based on the scale (case rate) and dynamics (changes in the number of cases) of COVID-19 incident rates^26^. According to this definition, a wave begins the first week with a weekly incidence rate above 70 cases per 100,000 inhabitants and with a positive growth incidence rate, while a wave ends the first week with a weekly incidence rate below 70 cases per 100,000 inhabitants and with a negative growth incidence rate for at least two consecutive weeks. Thus, for the Metropolitan Region, four study periods are identified, corresponding to four fully completed waves: a first wave from May 3, 2020 to July 26, 2020; the second wave between February 21, 2021, and July 11, 2021; the third wave from October 17, 2021 to November 21, 2021, and; the last wave covering the period between January 2, 2022 and April 24, 2022. To better capture the impact of COVID-19 deaths, two additional weeks were considered after the end of each wave to account for lags between the identification of cases and deaths.

For the analysis of health inequalities in the Greater Santiago area, the concentration index (CI) is computed. This index corresponds to a measure of economic inequality that has been adapted to measure health inequality. Conceptually, it is similar to the Gini index but it is derived from a bivariate distribution of health and social group ranking, and thus is not a measure of total inequality but captures the relationship between socioeconomic ranking and health^27–30^. The CI is derived from a concentration curve ($L(p)$) which is obtained by plotting the cumulative percentage of a health variable (y-axis) against the cumulative percentage of the population, ordered according to a socioeconomic variable (x-axis). In the hypothetical case that the health variable is evenly distributed in the population, a 45° line called the “line of equality” is drawn; on the contrary, when the distribution is unequal, the curve will lie above/below the line of equality, with a greater distance between the curve and the line meaning greater inequality in health. The CI is calculated as twice the area between the concentration curve and the line of equality^31^:

4 $$CI=1-2{\int }_{0}^{1}L(p)dp$$

All the previously mentioned scenarios can be summarized using the sign and magnitude of the CI. An index equal to 0 indicates equity in the health variable and an index with a negative/positive sign indicates that the burden of the disease is concentrated in the most economically disadvantaged/advantaged population, respectively. In addition, since the index fluctuates between -1 and 1, the closer to these limits the greater the inequity in health.

Analyzing inequalities by year and COVID-19 waves is useful to compare periods before and after the arrival of COVID-19 in Chile (years) as well as to analyze how these inequalities vary during the pandemic (waves). It should be noted that the analysis includes not only the cause of death of suspected COVID-19 cases (patients with symptoms or severe acute respiratory infection) and confirmed COVID-19 cases (patients who test positive in the RT-PCR test for SARS-CoV-2 or in the antigen test when it is a suspect case) but also the total number of deaths (from all causes). This is important to study how the COVID-19 pandemic containment measures and the classification of deaths impacted the rates of other causes.

### Data sources

The number of deaths by COVID-19 was obtained from public data provided weekly by the Department of Health Statistics and Information of the Chilean Ministry of Health^32^. This database records all deaths that have occurred in Chile since 2016, providing the primary cause of death classified according to the International Classification of Diseases (ICD10), in addition to the age, sex, and municipality of residence of the deceased. On the other hand, the population estimated for each municipality by age stratum was obtained from the National Institute of Statistics of Chile, using population projections based on the last national census of 2017^33^. The calculation of the municipality’s average income per household was obtained from the 2017 National Socioeconomic Characterization Survey (CASEN)^34^. Finally, the confirmed cases of COVID-19 came from the databases of the Chilean Ministry of Health available in a public repository of the Chilean Ministry of Science, Technology, Knowledge and Innovation^35^.

### Ethics approval

The study uses data obtained from open databases from various public institutions. Thus, the data have the characteristic of being anonymous, secondary and aggregated. In the Methods section we mention the different sources of information from which the data were obtained^32–35^. Thus, due to the characteristics already mentioned, this study was not submitted to an Ethics Committee.

## Results

Table 1 shows the mortality indicators for the Metropolitan Region. An increase in absolute PYLL for all causes in the last 4 years is observed, with the largest change occurring with the advent of COVID-19, growing 13.6% between 2019 and 2020; this rise is 4.5 times greater than the observed in the previous year. In fact, during the first pandemic year, there were 409,086 PYLL of which 90,734 were due to confirmed or suspected COVID-19, i.e., one-fifth of all absolute PYLL (22.2%). Similarly, to what was observed for the PYLL, the standardized mortality rate for all causes experienced an increase during the 4 years: the standardized rate in 2020 (8.5 deaths per 1000 inhabitants) represents an increase of 18.9% with respect to the previous year (7.1 deaths per 1000 inhabitants). It should be noted that despite the decrease in total the standardized mortality rate in 2021, the values of the mortality measures studied are still higher than the pre-pandemic levels. Continuing with the annual analysis, a decrease in both absolute PYLL and standardized mortality rate can be observed when excluding COVID-19 deaths. This does not necessarily mean an improvement in the other causes of death, as it is reasonable to consider other hypotheses, such as that excess deaths from COVID-19 would produce deaths in the population that would be expected in the short term, reducing the number of deaths for other causes.

**Table 1:** Summary of mortality indicators by periods in the Metropolitan Region.

Measure (weekly rate^a^)	Period							
	2018	2019	2020	2021	Wave 1	Wave 2	Wave 3	Wave 4
Duration in weeks	52	52	52	52	14	22	7	18
Stand. Mortality all causes	7.035 (0.135)	7.147 (0.137)	8.504 (0.164)	8.231 (0.158)	3.564 (0.255)	3.949 (0.180)	1.017 (0.145)	2.683 (0.149)
Stand. Mortality all causes except COVID-19	–	–	6.204 (0.119)	6.353 (0.122)	1.881 (0.134)	2.623 (0.119)	0.928 (0.133)	2.280 (0.127)
Stand. mortality total COVID-19	–	–	2.30 (0.044)	1.878 (0.036)	1.683 (0.120)	1.327 (0.0.060)	0.089 (0.013)	0.402 (0.022)
Abs. PYLL all causes	349,372 (6718.7)	359,908 (6921.3)	409,086 (7845.5)	412,769 (7937.9)	155,141 (11,081.5)	200,279 (9103.6)	50,737 (7248.1)	130,395 (7244.2)
Stand. PYLL all causes	49.564 (0.953)	49.598 (0.954)	55.223 (1.062)	54.623 (1.050)	21.181 (1.513)	26.600 (0.1.209)	6.668 (0.953)	16.945 (0.941)
Abs. PYLL all except COVID-19	–	–	318,352 (6105.4)	323,730 (6225.6)	87,554 (6253.9)	132,353 (6016.0)	47,533 (6790.4)	119,461 (6636.7)
Stand. PYLL all except COVID-19	–	–	42.559 (0.818)	42.605 (0.819)	11.750 (0.839)	17.455 (0.79)	6.236 (0.890)	15.470 (0.859)
Abs. PYLL total COVID-19	**–**	**–**	90,734 (1740.1)	89,039 (1712.3)	67,587 (4827.6)	67,926 (3087.5)	3204 (457.7)	10,934 (607.4)
Abs. PYLL confirmed COVID-19	**–**	**–**	71,107 (1363.6)	67,713 (1302.1)	52,098 (3721.3)	51,234 (2328.8)	2809 (401.3)	9873 (548.5)
Stand. PYLL total COVID-19	–	–	12.664 (0.244)	12.018 (0.231)	9.430 (0.0674)	9.145 (0.416)	0.432 (0.062)	1.475 (0.082)
Fraction PYLL total COVID-19	–	–	22.2%	21.6%	43.6%	33.9%	6.3%	8.4%

^a^Observation: the “weekly rate” is calculated dividing the indicator by the total number of weeks in its respective period.

To compare the different waves of COVID-19, a weekly rate of the mortality measures was calculated by dividing each of them by the weeks of duration of each wave. Considering the weekly rates of absolute PYLL for all causes, the first two waves are the most severe with 11,081.5 and 9,103.6 absolute PYLL per week, for which two-fifths (43.6%) and one-third (33.9%) are explained by COVID-19. It should be noted that with the evolution of the pandemic in Chile, the contribution of PYLL by COVID-19 in each wave is, in general, decreasing. A possible explanation for this result could be linked to the effectiveness of vaccination in the population. In the initial stage of the pandemic, no one had immunity to the virus due to the non-existence of a vaccine. However, as the pandemic progressed and vaccines were developed, an increasing proportion of the population became protected.

The advantage of analyzing both mortality rates and PYLL after the arrival of COVID-19 is that by considering the last one it is possible to differentiate whether different age groups were affected differently by the disease. Thus, due to the marked increase observed in both mortality measures in 2020, it is possible to mention that the population exhibited a higher number of deaths, and a large part of them correspond to young people (behavior similar to the first waves of COVID-19).

**Figure 1:**
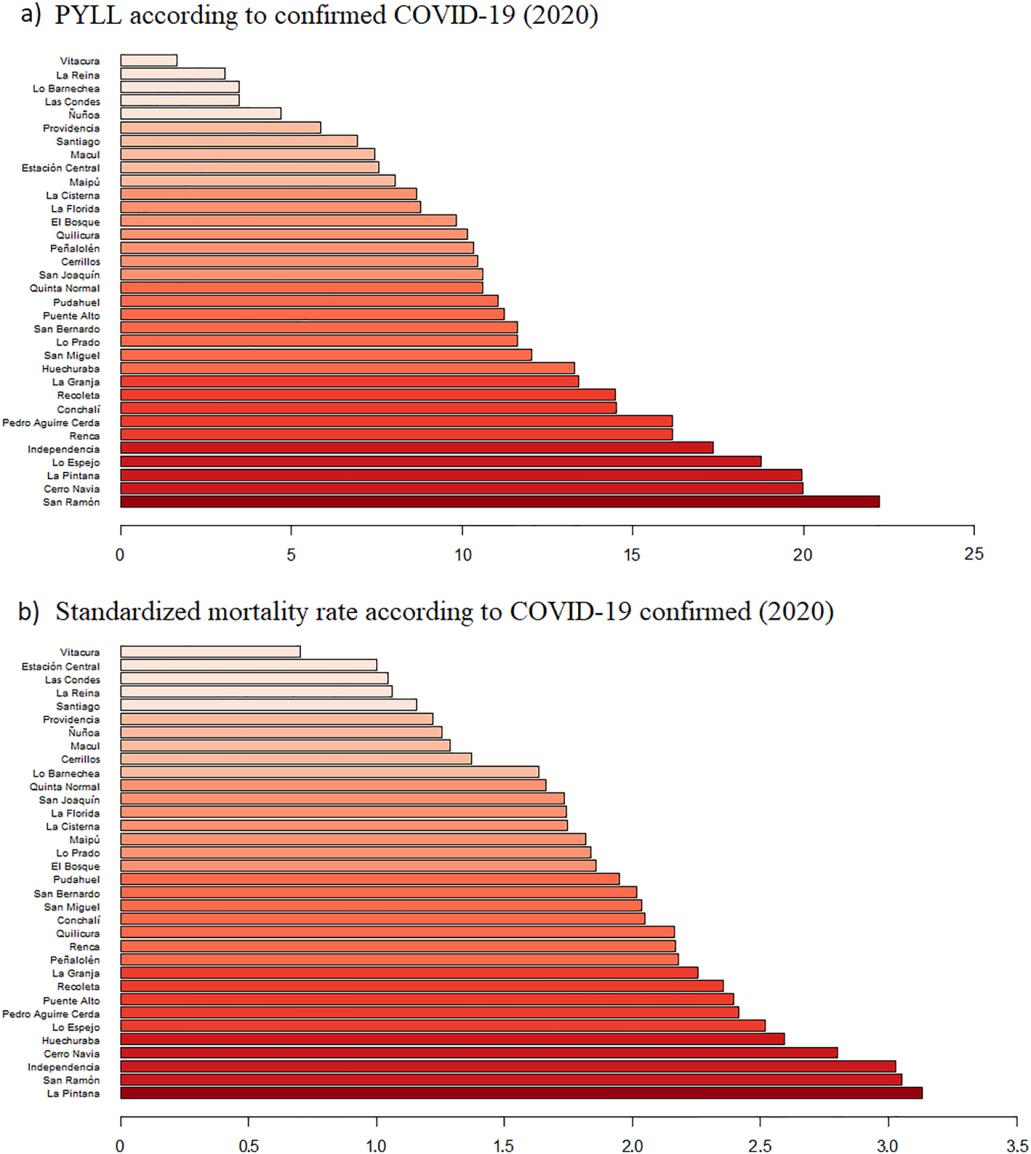
PYLL and mortality rate by COVID-19 confirmed (2020) in the municipalities of Greater Santiago.

Considering the previous analysis, it is interesting to observe the behavior of these variables at the municipal level. Figure 1 shows the standardized PYLL and mortality rates for COVID-19 confirmed in the first year of the pandemic (2020). As can be seen, the burden of the disease is unequal between municipalities, with the greatest differences for PYLL. Although the causes of inequity are multiple, we observe that municipalities with higher incomes—such as Vitacura, Lo Barnechea, and Las Condes—have lower values in mortality measures. On the other hand, the municipalities with the worst health outcomes are those with lower average income per household (more information about the socio-economic ranking of the municipalities in Supplementary Table 1).

Specifically, the confirmed COVID-19 standardized PYLL rate ranged from 1.66 in the least impacted municipality (Vitacura) to 22.22 in the most affected (San Ramón), which represents a ratio of 13.4. On the other hand, the standardized mortality rate ranged from 0.70 (Vitacura) to 3.13 (La Pintana), which represents a ratio of 4.5. This behavior is also observed considering all causes of death (see supplementary Fig. 1).

**Figure 2:**
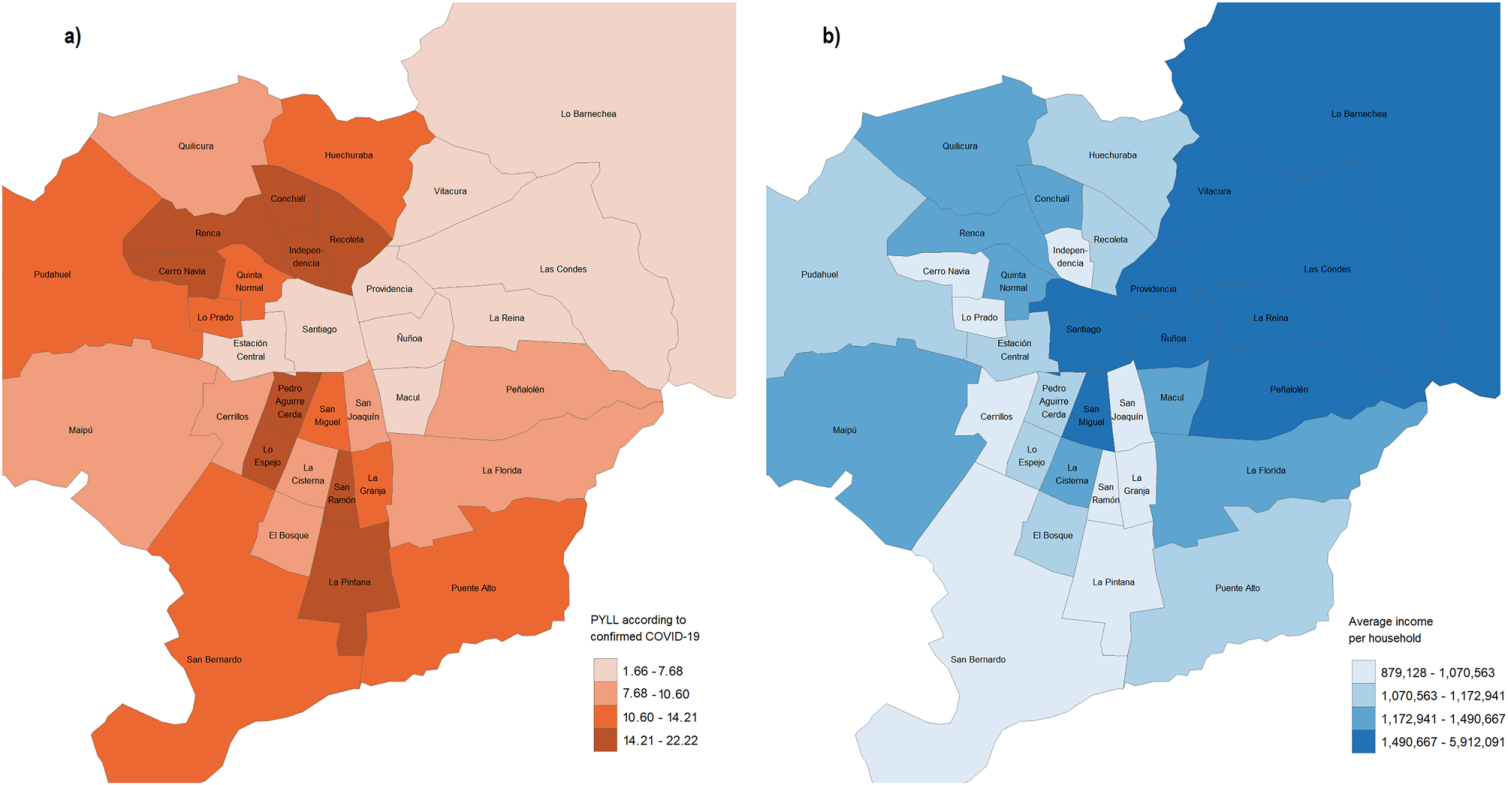
Spatial distribution of confirmed COVID-19 PYLL (2020) and average income per household.

The spatial relationship between PYLL and income is presented in Fig. 2. The figure on the left shows the spatial distribution of the PYLL rate for the first year of the confirmed COVID-19 pandemic, while the figure on the right shows the average income per household for each municipality. From them, it can be noted an inverse relationship between years of life prematurely lost and income (this relationship can also be observed in supplementary Figure N°2).

To quantify the inequality visualized in Figs. 1 and 2, the concentration index is calculated for all causes of death and, specifically, for COVID-19. These results are shown in Table 2. It is noteworthy that all values are negative, indicating that the burden of the disease is concentrated in the most economically disadvantaged municipalities of Greater Santiago. In addition, it should be noted that the inequities shown are more pronounced when estimating the burden of disease using PYLL, suggesting greater inequality in younger age groups.

**Table 2:** Concentration indexes for mortalities and PYLL according to cause of death and period.

Measure	Diagnostic group	Concentration index (CI)							
		2018	2019	2020	2021	Wave 1	Wave 2	Wave 3	Wave 4
Standardized mortality rates	All causes of death	−0.059	−0.058	−0.074	−0.064	−0.091	−0.079	−0.050	−0.055
	All except COVID-19	**–**	–	−0.059	−0.055	−0.067	−0.059	−0.045	−0.050
	COVID-19 confirmed and suspected	**–**	**–**	−0.112	−0.103	−0.116	−0.117	−0.095	−0.085
	COVID-19 confirmed	**–**	**–**	−0.115	−0.098	−0.119	−0.111	−0.090	−0.091
PYLL	All causes of death	−0.117	−0.112	−0.149	−0.122	−0.182	−0.126	−0.119	−0.129
	All except COVID-19	–	–	−0.138	−0.114	−0.168	−0.112	−0.117	−0.129
	COVID-19 confirmed and suspected	–	–	−0.186	−0.150	−0.200	−0.152	−0.137	−0.125
	COVID-19 confirmed	–	–	−0.187	−0.147	−0.202	−0.150	−0.110	−0.135

As can be observed, with the arrival of COVID-19 the PYLL and death rates indexes show an increase in inequality compared to the previous two years, which is present regarding whether or not we consider COVID-19 as the cause of death in 2020. The PYLL concentration index goes from −0.112 (2019) to −0.149 (2020) when considering all causes of death and to −0.138 when excluding COVID-19 deaths. Comparing the concentration indexes in the first year of the pandemic, more inequity is detected in COVID-19 deaths than in the remaining causes. On the other hand, in the second year of the pandemic a decrease in inequity is observed compared to the first year for all causes of death, returning to levels closer to those seen in pre-pandemic years; this decrease in inequity is also observed for COVID-19 deaths.

## Discussion

This study aims to characterize the burden of COVID-19 in terms of age-adjusted PYLL and mortality rates in the municipalities of the Greater Santiago, estimating its effect in different periods of the pandemic and analyzing potential inequities in the rates according to the municipality’s average household income. The results show that the first year of the pandemic (2020) is a breakpoint in the evolution of mortality indicators in the study population, with absolute PYLL and standardized mortality rate for all causes of death showing an increase of 13.6% and 18.9% over the previous year, respectively. Regarding deaths associated with COVID-19, it is possible to see that in both 2020 and 2021, one fifth of the total PYLL were contributed by this cause of death (22.2% and 21.6%). However, when examining the pandemic period by waves it is observed that the contribution of COVID-19 is, in general, decreasing. When PYLL is used, higher differences are observed in this indicator between municipalities than those observed when using standardized mortality.

The concentration index reflects inequality in mortality due to the average household income of the municipalities of Greater Santiago. In addition, the concentration index has a higher magnitude for PYLL than for standardized mortality. The PYLL differentiates deaths by the age at which they occur, then the greatest inequality is observed in younger age groups.

Although these findings are based on PYLL and concentration index indicators, they are consistent with those reported in other studies conducted in Chile based on age-adjusted mortality rates^10–12^ or excess mortality^13^ which also found a heterogeneous impact on mortality due to the Covid-19 pandemic, increasing pre-existing health inequities. In addition, the analysis between waves allows us to explore this result further, finding a stabilization in inequality as the pandemic progresses in Chile. For example, the first wave exhibits the greatest inequities for PYLL and mortality rates associated with confirmed COVID-19, and as new outbreaks emerge, the inequity assessed in each wave tends to attenuate (indexes closer to 0). One possible explanation is that the municipalities with lower average income were more affected in the initial stages of COVID-19, but through the strategies implemented to combat the advance of the epidemic—such as the massive vaccination plan—this inequity was reduced to some extent. Although there is consensus that the massive vaccination plan implemented in Chile was successful^4,36,37^, some authors have suggested possible social differences in the vaccine uptake^38^. In future research it would be relevant to explore how these differences may have influenced the results.

Other possible explanations are that the economic aid policies through vouchers allowed-in a certain way-a better compliance with quarantines of those who needed to make a living, or the possibility of a greater harvest effect in more deprived areas, where there is usually a higher density of disease and severe disease. Finally, it should be noted that despite this reduction in recent periods, these avoidable differences in the PYLL and mortality rates studied are far from being eradicated.

The study has limitations that should be considered during the analysis. First, there are temporal differences between the data used: while information related to deaths allows us to identify the impact on a certain municipality in a specific period, information related to population projections and average income per household used information from 2017. Thus, both standardized mortality rates and concentration indexes, which use these data, may present slight differences. It should be noted that population projections may be the most affected due to the increase in the migrant population in Chile in recent years^39^. However, there is no data source with more updated representative information. Additionally, a single threshold of 75 years has been used to calculate PYLL in order to be consistent with the national indicators that define life expectancy. However, it is important to acknowledge that this definition limits the scope of our findings, since it does not take into account that life expectancy varies with gender.

Secondly, this study uses the primary cause of death as a source for the analysis, since it is the only publicly accessible information in Chile. However, some primary causes of death could have not been identified as COVID-19, especially at the beginning of the pandemic when diagnostic tests were limited. In future research, it may be relevant to consider requesting information on multiple causes of death from the Chilean Ministry of Health.

Third, it should be noted that the results are descriptive of the evolution of health inequalities using aggregate data through the years and waves of COVID-19. Consequently, results can not identify causal effects and the ecological fallacy should be considered. Thus, although health inequality in PYLL/mortality rate due to COVID-19 attenuates as the pandemic evolved, future research is needed to assess the effect of public policies such as vaccination plan and economic aids on the mortality due to COVID-19.

Finally, it should be noted that this study focuses on the 34 urban municipalities of Greater Santiago with a large percentage of the Chilean population concentrated and significant socioeconomic and demographic heterogeneity. However, it is also important to recognize that other areas of the country could present a different behavior; it would be interesting to replicate the analysis in other geographic units. In this same sense, it would be important to extend the analysis beyond the critical moments of the pandemic (waves) by studying what happened between outbreaks of COVID-19, thus being able to have an even more complete understanding of the health inequalities presented in the pandemic period.

## Conclusions

This analysis shows the existence of health inequities (using the concentration index) in standardized mortality rates and years of life potentially lost by COVID-19, when comparing the urban municipalities of the Metropolitan Region according to average household income. It highlights that such inequity is more pronounced when estimating the burden of disease using PYLL, suggesting that the most economically disadvantaged municipalities were not only the most affected in terms of COVID-19 deaths but were also more unequally affected in deaths of younger groups.

We hope that this work will be useful to highlight the importance of revealing the distributive health effects present in an unequal country such as Chile. It will be useful both for decision-makers in the country and policy-makers around the world in the evaluation of future prevention and management strategies. Thus, the next generation of COVID-19 -or other emerging disease- policies should, for example, provide targeted support to more disadvantaged municipalities/groups. Along the same lines, the results may be relevant in the reformulation of the current health system.

Finally, we hope that this work will encourage the study of health inequalities based on other types of characteristics, beyond the economic, and in other contexts that help to minimize the negative effects caused by the pandemic.

### Supplementary Information

Supplementary Figure 1.

Supplementary Figure 2.

Supplementary Legends.

Supplementary Table 1.

## Data Availability

The COVID-related deaths data “Defunciones por Causa (actualización semanal)” that support the findings of this study are available from the open data of the Department of Health Statistics and Information [https://deis.minsal.cl/#datosabiertos]. The municipal projections of the population data “Estimaciones y proyecciones 2002–2035, comunas” are available from the open data of the National Institute of Statistics [https://www.ine.cl/estadisticas/sociales/demografia-y-vitales/proyecciones-de-poblacion]. The average income per household data obtained from the 2017 National Socioeconomic Characterization Survey (CASEN) is available in the following github repository [https://github.com/felipeelorrieta/Datasets/tree/master/casen]. Finally, the confirmed cases of COVID-19 data “Covid-19.csv” are available from the public repository of the Chilean Ministry of Science, Technology, Knowledge and Innovation [https://github.com/MinCiencia/Datos-COVID19/tree/master/output/producto1].

## Abbreviations

PYLL: Potential years of life lost
CI: Concentration index
