# Supplementary figures and images for: Inequity in mortality rates and potential years of life lost caused by COVID-19 in the Greater Santiago, Chile
Source: Sci Rep. 2023 Sep 28;13:16293. doi: 10.1038/s41598-023-43531-x (PMC10539509; doi:10.1038/s41598-023-43531-x)

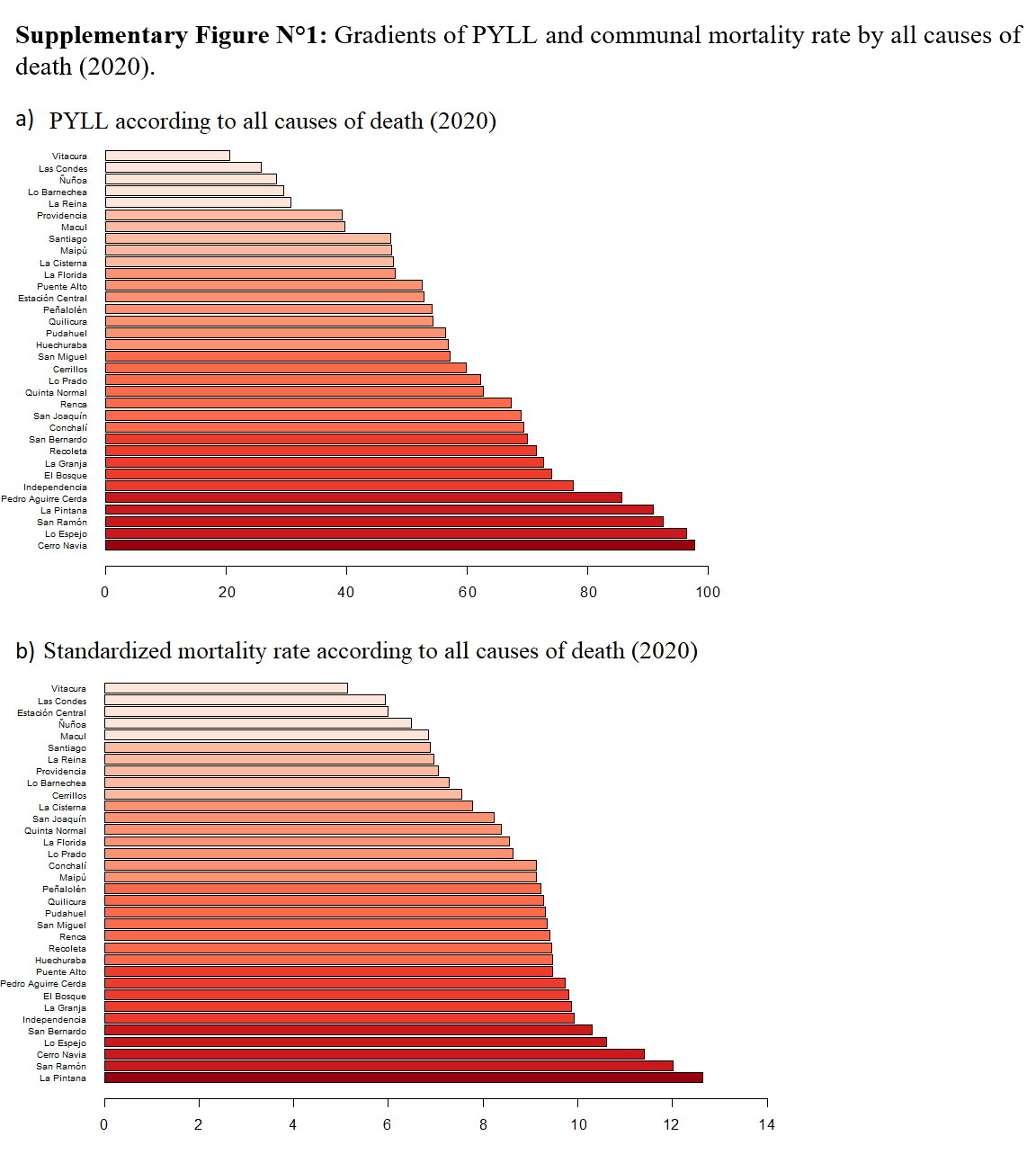

Supplement: Supplementary file 1 — Supplementary Figure 1. [file 41598_2023_43531_MOESM1_ESM.png]

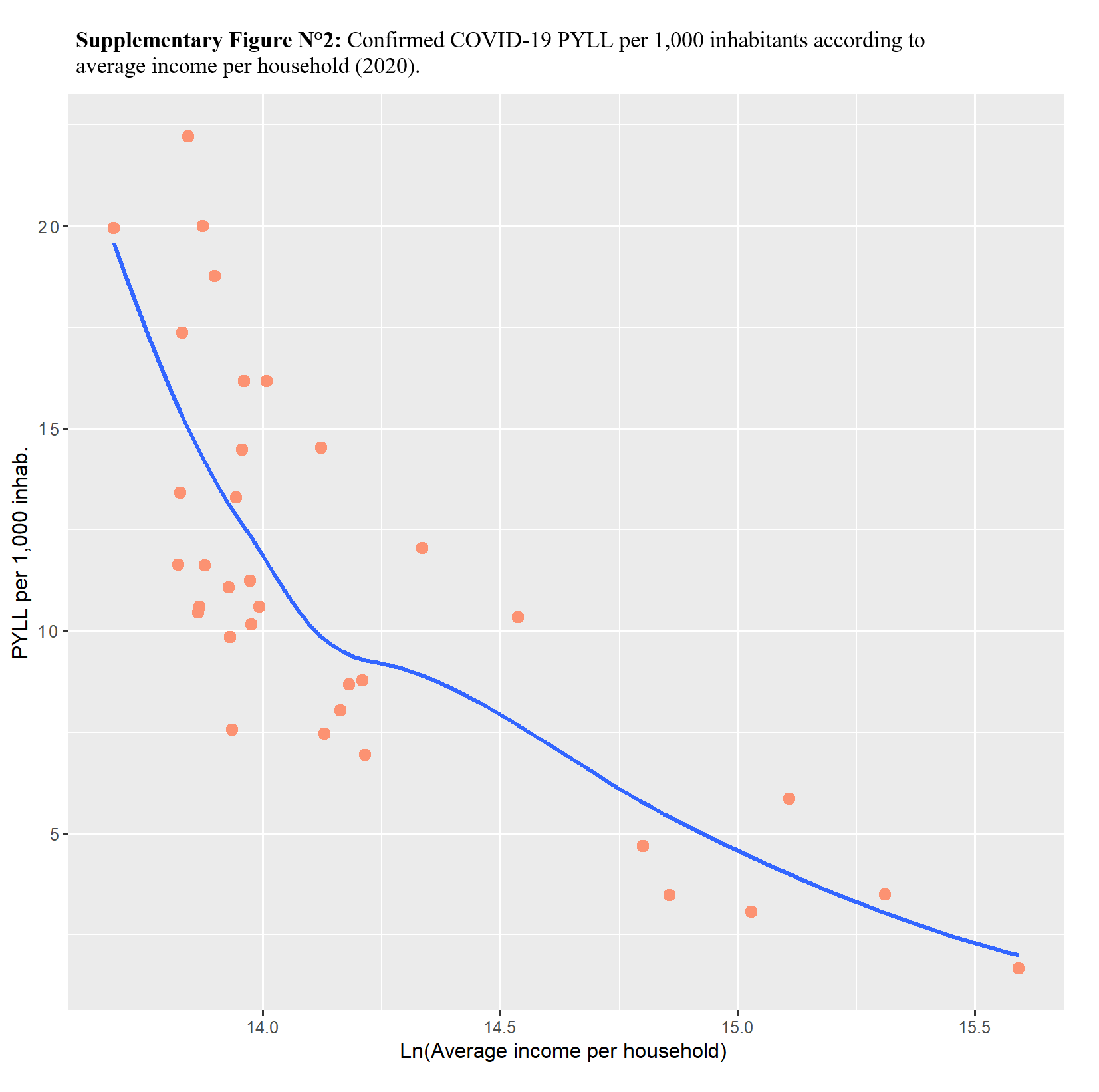

Supplement: Supplementary file 2 — Supplementary Figure 2. [file 41598_2023_43531_MOESM2_ESM.png]
