# Supplementary material for: Inequity in mortality rates and potential years of life lost caused by COVID-19 in the Greater Santiago, Chile
Source: Sci Rep. 2023 Sep 28;13:16293. doi: 10.1038/s41598-023-43531-x (PMC10539509; doi:10.1038/s41598-023-43531-x)
Supplement: Supplementary file 3 — Supplementary Legends. [file 41598_2023_43531_MOESM3_ESM.docx]

**Supplementary Figure N°1:** PYLL and mortality rate by all causes of death (2020) in the municipalities of Greater Santiago.

**Supplementary Figure N°2:** Scatter plot of confirmed COVID-19 PYLL per 1,000 inhabitants and average income per household (2020).
