# Supplementary material for: Inequity in mortality rates and potential years of life lost caused by COVID-19 in the Greater Santiago, Chile
Source: Sci Rep. 2023 Sep 28;13:16293. doi: 10.1038/s41598-023-43531-x (PMC10539509; doi:10.1038/s41598-023-43531-x)
Supplement: Supplementary file 4 — Supplementary Table 1. [file 41598_2023_43531_MOESM4_ESM.docx]

**Supplementary Table N°1**: Social Priority Index and average household income in the 34 municipalities of Greater Santiago (2017).

| Municipality | Social Priority Index | Average household Income (CLP 1000) |
| --- | --- | --- |
| Vitacura | 6.57 | 5,912 |
| Las Condes | 11.56 | 4,457 |
| Providencia | 27.13 | 3,646 |
| Lo Barnechea | 29.63 | 2,832 |
| La Reina | 32.67 | 3,365 |
| Ñuñoa | 37.67 | 2,677 |
| San Miguel | 49.67 | 1,684 |
| Quilicura | 54.88 | 1,174 |
| Maipú | 55.53 | 1,417 |
| Macul | 55.81 | 1,370 |
| Santiago | 56.06 | 1,493 |
| La Florida | 56.83 | 1,484 |
| Huechuraba | 57.95 | 1,138 |
| La Cisterna | 60.31 | 1,442 |
| Peñalolén | 62.45 | 2,059 |
| Pudahuel | 62.61 | 1,120 |
| Puente Alto | 63.47 | 1,172 |
| Estación Central | 64.54 | 1,128 |
| Cerrillos | 65.23 | 1,050 |
| Pedro Aguirre Cerda | 67.69 | 1,156 |
| Renca | 68 | 1,212 |
| Independencia | 68.16 | 1,015 |
| Quinta Normal | 68.16 | 1,195 |
| La Granja | 68.57 | 1,011 |
| Recoleta | 70.08 | 1,151 |
| El Bosque | 70.87 | 1,123 |
| Lo Prado | 71.61 | 1,007 |
| San Bernardo | 72.17 | 1,065 |
| San Joaquín | 74.52 | 1,053 |
| Conchalí | 75.87 | 1,360 |
| San Ramón | 78.32 | 1,028 |
| Cerro Navia | 79.18 | 1,060 |
| Lo Espejo | 79.46 | 1,087 |
| La Pintana | 80.72 | 879 |
| The Social Priority Index (SPI) is an indicator that measures the relative level of development achieved by each municipality. A small/high value of the SPI indicates low/high social priority. | | |
